# With COVID Comes Complexity: Assessing the Implementation of Family Visitation Programs in Long-Term Care

**DOI:** 10.1093/geront/gnac175

**Published:** 2022-12-03

**Authors:** Stephanie A Chamberlain, Grace Warner, Melissa K Andrew, Mary Jean Hande, Emily Hubley, Lori E Weeks, Janice M Keefe

**Affiliations:** Faculty of Nursing, College of Health Sciences, University of Alberta, Edmonton, Alberta, Canada; School of Occupational Therapy, Faculty of Health, Dalhousie University, Halifax, Nova Scotia, Canada; Department of Medicine (Geriatrics), Dalhousie University, Halifax, Nova Scotia, Canada; Department of Sociology, Trent University, Peterborough, Ontario, Canada; Nova Scotia Centre on Aging, Mount Saint Vincent University, Halifax, Nova Scotia, Canada; School of Nursing, Faculty of Health, Dalhousie University, Halifax, Nova Scotia, Canada; Nova Scotia Centre on Aging, Mount Saint Vincent University, Halifax, Nova Scotia, Canada; Department of Family Studies and Gerontology, Mount Saint Vincent University, Halifax, Nova Scotia, Canada

**Keywords:** Family/friend caregivers, Implementation science, Nursing home, Pandemic, Visitation policies

## Abstract

**Background and Objectives:**

Coronavirus disease 2019 (COVID-19) pandemic visitor restrictions to long-term care facilities have demonstrated that eliminating opportunities for family-resident contact has devastating consequences for residents’ quality of life. Our study aimed to understand how public health directives to support family visitations during the pandemic were navigated, managed, and implemented by staff.

**Research Design and Methods:**

Guided by the Consolidated Framework for Implementation Research, we conducted video/telephone interviews with 54 direct care and implementation staff in six long-term care homes in two Canadian provinces to assess implementation barriers and facilitators of visitation programs. Equity and inclusion issues were examined in the program’s implementation.

**Results:**

Despite similar public health directives, implementation varied by facility, largely influenced by the existing culture and processes of the facility and the staff understanding of the program; differences resulted in how designated family members were chosen and restrictions around visitations (e.g., scheduling and location). Facilitators of implementation were good communication networks, leadership, and intentional planning to develop the visitor designation processes. However, the lack of consultation with direct care staff led to logistical challenges around visitation and ignited conflict around visitation rules and procedures.

**Discussion and Implications:**

Insights into the complexities of implementing family visitation programs during a pandemic are discussed, and opportunities for improvement are identified. Our results reveal the importance of proactively including direct care staff and family in planning for future outbreaks.

## Background and Objectives

The coronavirus disease 2019 (COVID-19) pandemic has had a devastating toll for those in residential long-term care (LTC) homes (also known as nursing homes and continuing care facilities) in Canada. To keep residents and staff safe from COVID-19, public health measures were put in place early in the pandemic restricting visitation with residents. While restrictive visitation measures aimed to limit the spread of the COVID-19 virus, the prolonged separation from family and friend caregivers (hereafter named family) had a tremendous negative effect on the mental health of residents, including increased feelings of loneliness (Huber & Seifert, 2022), increased depressive symptoms (Hugelius et al., 2021), and other cognitive and behavioral problems (Kyler-Yano et al., 2022; Van der Roest et al., 2020). Family plays a critical role in LTC, often assisting with feeding, grooming, and mobility. Strong interrelationships among residents, family, and staff are essential to resident quality of life (Barken & Lowndes, 2018; Barken et al., 2017; Baumbusch & Phinney, 2014; Zimmerman et al., 2013).

COVID-19 highlighted preexisting deficiencies in LTC homes. Increased resident acuity and stagnant staffing ratios are persistent issues in the LTC sector (Chamberlain et al., 2019; Hoben et al., 2019), and restrictions had significant negative consequences for LTC home staff and residents. Staffing shortages were prevalent because of staff illness and pandemic-instigated rules limiting staff to working at only one home, and staff were often performing additional tasks because of limited external supports (Backhaus et al., 2021; Gray et al., 2021; Low et al., 2021). When community transmission lessened, Canadian provincial officials allowed on-site visitation, initially outdoors, and subsequently through “support visitations,” where a designated person was allowed access to the LTC home to assist with care tasks. Provinces varied in their programs and policies for support visitation, and this variation persisted throughout the multiple waves of the pandemic (Freeman, 2021). Differential implementation of support visitation programs may lead to unintended consequences for the resident, family, and LTC home staff, especially given their lack of involvement in policy decisions and implementation strategies, which may exacerbate preexisting inequities (Siegel & Young, 2021). It is critical to understand factors that support adoptability, implementability, and sustainability of the intervention; and the anticipated outcomes of support visitations for residents, family, and staff. The purpose of this project was to understand the staff’s perspective of implementation, execution, and impact of support visitation programs in LTC homes during the COVID-19 pandemic.

## Study Design and Methods

### Theoretical Framework

The project was guided by the Consolidated Framework for Implementation Research (CFIR) Version 1.0 (Damschroder et al., 2009). There are numerous implementation science theories, models, and frameworks (Nilsen, 2015), and the CFIR was chosen because it assesses health system-level factors that affect the successful implementation of an intervention (Kirk et al., 2016; McIsaac et al., 2018). It can be used in research to explore barriers and facilitators to implementation (formative evaluation) and the extent to which implementation was perceived to be effective. The CFIR has five domains and 39 constructs that are determinants of the successful implementation of innovative programs (Supplementary Table 1). The domains assess (a) intervention characteristics, (b) outer setting, (c) inner setting, (d) characteristics of individuals, and (e) process. Recent research extending the CFIR aimed to elaborate and clarify the types of outcomes used with the CFIR, specifically distinguishing implementation and innovation outcomes (Damschroder et al., 2022). Implementation outcomes assess the success or failure of implementation and are classified as anticipated outcomes and actual outcomes. Innovation outcomes are indicators of innovation success or failure and their impact on key constituents, including key decision makers, innovation recipients, and innovation deliverers (Damschroder et al., 2022). In the most recent CFIR addendum, the higher order or parent constructs of implementation climate and implementation readiness were reclassified as antecedent assessments, the lower order or child constructs within implementation climate and readiness remain under the inner setting domain (Supplementary Table 1). The CFIR domains and constructs were used to structure interview guides, generate interview questions, and for deductive analysis. The purpose of this study was to identify barriers and facilitators in the actual implementation process of support visitations in LTC from the perspective of the LTC staff.

### Setting and Sample

This qualitative study was conducted in six LTC homes in two eastern Canadian provinces (Nova Scotia: *n* = 4; Prince Edward Island: *n* = 2). The term for a designated visitor in Nova Scotia is “Designated Caregiver” and in Prince Edward Island it is “Partner in Care”; here, we refer to both as designated caregivers. At different times during the pandemic, both provinces allowed for nondesignated family caregivers to play a supportive role (referred to as nondesignated caregivers). A timeline of events related to support visitation programs in the two provinces is shown in Supplementary Figure 1. The LTC homes in our sample were varied in organizational structure, layout, design, building age, and bed size. Only one LTC home experienced a case of COVID-19 during the pandemic, which occurred prior to this study (Supplementary Table 2).

### Participants and Recruitment

LTC home staff participants were implementation staff (involved in the initial design and ongoing implementation of the program) and direct care staff (not involved in the design and oversight of the program but supported residents with and without a designated caregiver). LTC home administrators (or their equivalent) assisted with study recruitment by identifying implementation staff and direct care staff and asking if they agreed to be contacted by study staff. All participation was voluntary. Inclusion criteria were being members of any one of the groups of focus (implementation staff or direct care staff), an interest in participation, and the ability to be interviewed in English.

### Data Collection

The CFIR domains and their constructs guided our interview data collection and analysis. The CFIR domains represented the following entities in our study: Intervention = support visitation; Outer setting = Province/region; Inner setting = LTC home; Individuals = Implementation staff and direct care staff; and Process = Process of implementing support visitation. Interview guides were designed to identify the individual (staff), structural (physical space and location), organizational (culture and leadership), and policy (organizational and provincial/regional) contexts affecting implementation processes. Our primary focus was on process factors; however, interviews did discuss implementation outcomes, including the extent to which the program was currently being delivered (implementation) and perceptions of how it may be delivered over time (sustainment). Sex, gender, and other factors (i.e., age, socioeconomic status, culture, and sexuality) were considered in the design and analysis to ensure that we included gender-diverse perspectives and addressed considerations of these subpopulations. For example, interviewers asked participants to reflect on how aspects of the program might have differentially influenced residents and families based on a variety of factors, including age, sex and gender, financial status, race and ethnicity, language, and family structure (see Semi Structured Interview Guide in Online Supplementary Material).

A team of five research assistants were trained by study investigators to conduct the interviews, including procedures for data collection and analysis. Study investigators were from relevant disciplines (i.e., gerontology, sociology, geriatrics, and implementation science) with expertise in qualitative interview methods.

Research staff sent informed consent documents to participants who indicated an interest in being interviewed. The interviewer reviewed the informed consent with participants before the interview and answered any questions. All interviews were completed remotely by videoconference or telephone, and audio was recorded. LTC homes received a $2,000–$3,500 honorarium for replacement staff depending on the LTC home’s bed size. LTC home staff were not directly compensated but participated in the study during work hours.

### Interview Guides

Interview guides were developed using the CFIR constructs (Online Supplementary Material). Questions were added to assess participant demographic characteristics (e.g., age, gender, education, country of origin, and ethnic identity), work characteristics (position and tenure), resident and family member inequities (e.g., sex, gender, financial resources, family dynamics, and language), and broader implementation issues specific to each participant group. Implementation staff were asked about the program implementation process, barriers, and enablers; and how program implementation might have affected residents, family, and staff. Direct care staff, who were not involved with the design or set-up of the program, were asked about their experiences implementing support visitation programs and their observations of the impact on residents. The guides were developed by study investigators and piloted with study partners.

### Analysis

We analyzed interview data using the rapid assessment process (RAP; Beebe, 2001). RAP is an intensive, team-based approach to qualitative data analysis that uses triangulation, iterative data collection, and iterative data analysis to quickly gain “the insider’s perspective.” RAP is appropriate for this study because it is used in studies with a short timeline (our study was completed in 1 year, inclusive of ethics, recruitment, data collection, analysis, and dissemination of results to project partners) and when timely findings are needed to inform project partners. A rapid qualitative analysis approach using CFIR is a credible and rigorous approach that allows the research team to provide rapid feedback to health system partners (Nevedal et al., 2021). RAP analysis typically includes initial deductive coding and sorting, followed by inductive coding, as needed. We examined sex, gender, and other factors in both the deductive coding phase (examining the responses to the specific questions and probes) and inductive coding phase (examining more broadly for issues of equity that were not identified in our probes). The following describes our data collection and analysis processes.

1. Before data collection, an Excel file was generated for each interview. Rows reflected individual participants and columns reflected interview questions grouped into broad categories aligned with various CFIR constructs: staff background, implementation processes, implementation experiences to understand multilevel barriers and facilitators, impact, resources (i.e., human, equipment, and financial costs), and overall interviewer comments on the interview. Subcategories within each category further refined the data.
2. After the interview, the same research assistant documented key findings in each category. If necessary, audio recordings were revisited to capture nuances/context and verbatim responses; full responses or excerpts were transcribed to support analysis.
3. To ensure the consistency and usability of the Excel document, a second research assistant reviewed the recorded interviews and documented any additional findings. The two documents were compared and consolidated, so the key findings represented the perspectives of both research assistants.
4. At least one investigator reviewed the research assistants’ analyses, and discrepancies were discussed with the team. Differences were resolved by consensus, and a final consolidated summary was completed for each interview participant.
5. Completed interview summaries were transferred to a matrix of all interview participants, with participants in rows and categories/subcategories in columns.
6. Once the matrix contained all participant interview data, two research assistants separately conducted a content analysis using a structured sequence within each subcategory. Codes were first developed deductively (reflecting the CFIR constructs), and additional codes were developed inductively over time to ensure they reflected participants’ perspectives (Hsieh & Shannon, 2005; Krippendorff, 2004). All coding was reviewed and challenged by investigators.
7. After subcategory coding was completed, coding was summarized for each subcategory with exemplary quotes and details to reflect varying interviewee responses. Subcategory codes were compared, and similar codes were aggregated to develop initial themes for each subcategory. Subcategories were compared and synthesized to identify overall themes for each participant category.
8. Themes were examined by the full team, presented to facility stakeholders for feedback, and finalized.

All study data were deidentified. Participants were assigned a research identification number that was linked to their name and contact information in a separate password-protected file.

### Ethics

This study was approved by Mount Saint Vincent University (REB # 2020-129), Dalhousie University (REB # 2021-5456), University of Alberta (REB # Pro00108265), University of Victoria (REB # 21-0003), and Prince Edward Island Health Authority.

## Results

We interviewed 54 LTC home staff (32 implementation staff and 22 direct care staff). Staff were mostly female (*n* = 45, 84%), 44 years in age (standard deviation [*SD*] = 11), and born in Canada (*n* = 48, 89%; Table 1). Staff responsible for implementing support visitation programs came from a variety of roles, including recreation (*n* = 7, 22%), managers and directors (*n* = 12, 37%), and LTC assistants (*n* = 6, 19%). Direct care staff were primarily continuing care aides (*n* = 20, 91%) and resident care workers (*n* = 2, 9%).

**Table 1.** Characteristics of Staff Member Participants

Characteristics	Implementation staff (*n* = 32)	Direct care staff (*n* = 22)	Total (*N* = 54)
	*N* (%) or *M* (*SD*)	*N* (%) or *M* (*SD*)	*N* (%) or *M* (*SD*)
Province			
Nova Scotia	22 (69%)	14 (64%)	36 (67%)
Prince Edward Island	10 (31%)	8 (36%)	18 (33%)
Age range (years)	45 (11)	43 (12)	44 (11)
<35	6 (19%)	9 (41%)	15 (28%)
35–44	7 (22%)	2 (9%)	9 (17%)
45–55	14 (43%)	8 (36%)	22 (40%)
>55	5 (16%)	3 (14%)	8 (15%)
Gender			
Male	4 (12%)	5 (23%)	9 (16%)
Female	28 (88%)	17 (77%)	45 (84%)
Highest education level			
High school	3 (9.5%)	0 (0%)	3 (5%)
College/diploma	8 (25%)	15 (68%)	23 (43%)
Some university/university	18 (56%)	7 (32%)	25 (47%)
Master’s degree	3 (9.5%)	0 (0%)	3 (5%)
Country born			
Canada	30 (94%)	18 (82%)	48 (89%)
Outside Canada	2 (6%)	4 (18%)	6 (11%)
Ethnic group^a^			
White	28 (88%)	18 (82%)	46 (85%)
Non-White (Asian, South Asian, East Asian, Metis, and not specified)	4 (12%)	4 (18%)	8 (15%)
Position at LTC home			
Recreation	7 (22%)	2 (9%)	9 (17%)
LTC assistant	6 (19%)	0 (0%)	6 (11%)
Management	12 (37%)	0 (0%)	12 (22%)
Continuing care assistant	0 (0%)	20 (91%)	20 (37%)
Other	7 (22%)	0 (0%)	7 (13%)
Years working at LTC home	9 (9)	6 (6)	8 (8)

*Notes*: LTC = long-term care; *SD* = standard deviation.

^a^Participants were asked to indicate in their words which ethnic group they most identified with.

Guided by the CFIR, our analysis resulted in two overarching themes (external innovation; organizational context) related to barriers and facilitators to the implementation of support visitation. Within these themes, many of the CFIR constructs are represented (Table 2). We describe the themes later.

**Table 2.** Findings and Relevant CFIR Domains, Constructs, and Their Impact on Implementation

CFIR domain	CFIR construct	Barrier	Facilitator
Theme 1: External innovation			
Intervention characteristics	Intervention source (external)	No proactive engagement with LTC facilities, short timelines, and frequent changes	Province accountable for directive rather than staff
	Adaptability	Inability to adapt the overall provincial policies	Facilities’ ability to adapt implementation processes to fit their organization
	Trialability	Inability to trial the directive before implementation	
Characteristics of individuals	Knowledge and beliefs about the intervention	Lack of staff access to knowledge of the directive, unsure which residents benefited	Staff believed in the benefits of directive
Inner setting	Structural characteristics	Lack of physical space in small facilities for visitations	Large spaces where distancing could take place
	Available resources	Lack of additional resources from the provinces	Additional provincial funding to hire staff
	Access to knowledge and information	Lack of organizational knowledge on how to incorporate directive into the existing work flow	
	Relative priority		Facilities and staff believed the directive was beneficial
Theme 2: Organizational context			
Outer setting	Patient needs and resources	Staff inability to balance resident needs with directive mandates	Families supported the initiative
Inner setting	Culture	Hierarchical structure limiting communication	Supports teamwork, strong organizational leadership, and values staff communications
	Learning climate	No time for reflective thinking and evaluation	Staff feeling valued and part of the process
	Goals and feedback		Organizational goals communicated to staff and staff feedback is gathered
	Relative priority		Facilities and staff believed the directive was beneficial
	Leadership engagement	Lack of day-to-day engagement from organizational leadership	
	Available resources	Lack of facility money, staff, and time to implement directive	
	Access to knowledge and information	Facilities poor at helping staff and families access information about changing processes	Frequent and varied communications with staff

*Notes*: CFIR = Consolidated Framework for Implementation Research; LTC = long-term care.

### Theme One: External Innovation

The visitation programs were an external innovation mandated by provincial governments in all provincial/regional LTC homes. According to our participants, the external nature of the innovation led to issues during implementation. Provincial and regional governments did not proactively engage with LTC home leadership and program implementors before announcing the program. Although LTC home staff believed family member visitation was critical to resident well-being, LTC homes were given no guidance on how to best implement the directive. The externally driven initiative was viewed by some staff as easing their burdens. They were not responsible for developing the program or fielding potential criticisms; they only managed the in-facility program logistics. However, staff could not easily adapt to the overarching support visitation policy throughout the pandemic because program expectations were set by the province and not tailored to individual LTC homes. In many cases, LTC staff did not receive adequate information and did not have access to the necessary knowledge for program implementation. Program logistics and strategies were left to LTC staff:

They make all these big statements to the media and then they send us hundreds and hundreds of pages of notes on things that we can follow but [the Chief Medical Officer of Health; Ministry of Health] always leaves the decision back, in the end, to the facility. So, [Ministry of Health] says you can bring designated caregivers in, but you figure out the day, the time, how you’re gonna do it, where you’re gonna do it, how you’re gonna set it up—they don’t tell you any of that stuff. (Implementation Staff, Director of Recreation, Nova Scotia)

The expected speed of change was a particular challenge during program implementation, including the province’s frequent policy changes and the timing of announcements for these changes. Staff appreciated that the province allowed them to determine how the program would be implemented in their facility, but staff needed time to create policies and procedures for their LTC home. The expectation for quick program implementation meant that direct care staff did not have time to trial implementation processes or identify how they could be improved. Some implementation staff said they would have consulted families, as they have in past program development, but the top–down nature of the provincial mandates coupled with time constraints made this impossible. In most cases, LTC home staff described having limited time to prepare for implementation and were often frustrated with unexpected government announcements and the fast pace of change. Provincial representatives would typically announce changes through media releases like televised public press conferences (e.g., an additional person could apply as a designated caregiver) and, at the same time, inform LTC operators and LTC homes. LTC homes would then have to implement the revised policies and procedures in a very short time frame, usually within days. The public often knew about changes before the LTC homes could develop an implementation strategy. Family members would arrive at the LTC home the day after a press conference expecting changes to be already implemented. LTC homes would sometimes not be entirely aware of the scope of changes, and staff would scramble to adapt their activities to match the new directives:

With some changes, they [family] would learn about them from social media before the care home knew or informed staff. At times, the staff would apologize to the PiC [designated caregiver] for not knowing what they were talking about when they heard of changes from the news. We’re the last ones to know but the first ones who need to deal with it. (Direct care staff, Resident Care Worker, Prince Edward Island)

We would find out this information at the same time the public would. Often, well every time, we are, like the families, the public are finding out before us and then we would get inundated with phone calls and requests, you know they’re feeling a sense of urgency, they want to get in to see their loved ones. We often found ourselves having to say we know, we have the information, yes, it’s going to come, but we need to have our operations in place first—something along those lines—something like “we’re not quite ready.” (Implementation staff, Nurse Manager, Prince Edward Island)

Although the program was externally mandated, additional resources from the external source were not consistently provided. Resource constraints were critical barriers to implementation. For many LTC homes, implementation was made more challenging by limited facility resources, primarily limited staffing capacity, and lack of available space. These LTC homes had to determine where visits could happen in constrained spaces, while simultaneously following local public health orders that mandated physical distancing. LTC homes with enough open space (e.g., large common room and private rooms) did not need to limit the number of visitors, but LTC homes with limited or small communal spaces had to restrict visitors to one at a time. Visiting hours were also affected by space availability. The number of visitors allowed into the facility and flexibility in visiting hours were also largely dictated by available staffing resources. For example, some LTC homes could accommodate visitors from 9 a.m. to 6 p.m., other LTC homes were limited to three designated visiting hours and a maximum number of people at one time (e.g., 11 a.m. to 1 p.m. and 2 p.m. to 4 p.m. with three visitors per unit). These capacity-driven decisions affected the number of family and residents who could benefit from the program:

If you’re [the government] going to ask people to do something they should provide the resources that are needed to do it. We’re short staffed all the time but [we are] expected to roll out this plan without extra staff. (Implementation staff, Nurse Manager, Prince Edward Island)

Implementation staff described persistent staff shortages as a barrier to implementation, whether preexisting shortages or shortages due to redistribution of tasks during the pandemic. Some staff were moved from their normal roles into a different role necessitated by the visitation program (i.e., screening designated visitors at the door), leaving other departments short of staff. For example, the recreation department in one LTC home was entirely redirected to implementation efforts, affecting programming for residents:

We need more people … we struggle every day how we’re going to cover that person screening at the door … other services have to cut back in order to have that person there, like our recreation services have to cut back because that recreation person has now got to be at the door. (Implementation Staff, Nurse Manager, Prince Edward Island)

Additional provincial funding during the pandemic allowed several facilities in Nova Scotia to create a new position, called a LTC assistant, for more staffing capacity and to help with the additional activities generated by the visitation program. Many staff credited this additional funding and the hiring of LTC assistants for their ability to implement the program. Referencing the implementation of the program, one direct care staff stated “[We] could not have made it happen without the LTC assistants,” with another staff member saying that the LTC assistants were the “superstars of the program.” LTC staff indicated that the visitation program needed dedicated staff, such as a full-time manager, because allocating tasks to existing staff was not sustainable:

At the beginning there was a lot of teamwork. They had a COVID schedule and people were always willing to show up and help where needed. Now it’s just a mad scramble to find people to work “cause we”re all burnt out. At the beginning we thought ok it won’t last very long but now it’s getting monotonous, it’s just ready to be over. (Direct care staff, Continuing Care Assistant, Nova Scotia)

Although the visitation program came from an external source, staff welcomed the initiative, recognizing the benefits of family involvement and support visitation to residents. Staff believed that family were critical to the physical and mental well-being of residents and immediately recognized the need for a visitor program to facilitate family entry to the LTC home after restrictions were introduced. Staff buy-in was critical for timely implementation. Many staff initially agreed to adapt their roles, add to their responsibilities, and take on additional shifts to ensure program implementation (i.e., that designated visitors could enter the LTC home). One staff member said, “You can see the difference in a resident when somebody’s continuously coming. We might be their person but we’re not their family at the end of it” (Direct care staff, Continuing Care Assistant, Nova Scotia).

While most staff believed that the program offered significant benefits to all residents, there was variation in the degree of perceived benefit based on resident cognitive impairment. Some direct care staff felt that residents without dementia, or with only mild cognitive impairment, benefited most from visitors. The following quotes indicate the different perspectives on the program’s impact on residents with and without dementia:

I do think people with dementia … people might make the comment “well they don’t know” but actually they do know. If somebody’s been married to somebody for 50 years, even though they have dementia that’s the one face in the world that they are gonna recognize or that they’re gonna look for … so I think that for most people, the ones with dementia, it’s [the program] been really significant. (Implementation staff, Director of Education, Nova Scotia)

The ones that are aware of what is going on around them, they benefit from it [the designated caregiver program]. They really benefit. The other residents that really aren’t aware of what’s going on around them, you know, what are they understanding? Do they understand? You know? But for the ones that are aware there is major impact, you can see the difference in them … you know it’s positive. (Direct care staff, Continuing Care Assistant, Nova Scotia)

### Theme Two: Organizational Context

LTC staff had to plan and implement the visitation program while balancing resident safety and resident well-being. Staff described needing to balance the need to keep residents safe (e.g., requiring families to wear personal protective equipment, and adhere to public health protocols) with trying to be flexible with how and when families could visit. Staff understood the importance of visitors, but as one LTC assistant described, “we are to blame if someone gets sick.” Available resources were critical to implementation efforts. Additional staff resources, often through the LTC assistant funding, were described as one of the most beneficial resources to implementation, ensuring that there were dedicated staff for implementation. Unfortunately, not all homes were able to hire staff specifically to implement the program. Staff responsible for implementation varied in each home; in some LTC homes, recreation staff were primarily responsible, other homes allocated the responsibility to administration and management, while a few had support from their corporate office. Responsibilities for booking visits and screening at the door also varied between homes; one home hired outside security to do screening, others used recreation staff, and some asked visitors to self-screen.

Organizational context factors were both a barrier and facilitator to implementation. Communication was a persistent challenge for staff, particularly for part time or casual staff who were not on site regularly to receive updates. A lack of communication from management to direct care staff (e.g., not timely, not clear, or not enough updates on changes) was identified as a barrier to timely implementation and often was present in the facility prior to implementation. Insufficient communication had a ripple effect on families, who were often unaware of ever-changing policies or if they were not following the current rules. Direct care staff would have to take the unpleasant task of monitoring and reminding families of the rules. Fractured communication also meant that staff were not always up to date on program changes, and families would get different messages depending on who they asked. One direct care staff member said she once greeted a designated caregiver and told him that, based on her current understanding, he was not allowed to enter; another staff member intervened saying “no, he is allowed.” Direct care staff indicated they needed timely education, regular updates, and clear communication about the rules to effectively implement the program. Implementation staff identified some ways that communication facilitated implementation efforts. Frequent team meetings were ways that staff could plan or discuss the program. Communication from upper management was viewed as helpful when they could obtain answers from the staff at a higher and more informed level. A few implementation staff said that question-and-answer periods with the provincial Department of Health or the regional Health Authority were helpful.

They were really good at putting out the notices with memos because we are a 24 hour facility so we don’t always get to see our managers or management staff, so a lot of it was email based cause if you were on the evening or night shift, even though partners in care weren’t here you needed to be aware of what changes were happening … so the memos were very important to keep us all up to date … during the day time it was a lot of verbal communication. (Direct care staff, Resident Care Worker, Prince Edward Island)

The existing culture and processes of the facility, including leadership and teamwork, positively influenced implementation. A sense of teamwork and collaboration meant staff were willing to pivot from existing roles to new activities necessary for program implementation. Teamwork among LTC staff was facilitated if staff were open, positive, collaborative, and encouraged input. Staff described support from upper management as showing appreciation through morale boosters, patience, providing good direction, and being responsive to questions.

[Support from management] has been positive, yes. I think everyone’s stressed and … they’re all doing the best that they can do, but they’re trying to be as positive as they can under the circumstances while still making very important decisions … the wrong decision can affect the whole building. They have a lot on their shoulders. (Direct care staff, Continuing Care Assistant, Nova Scotia)

I would have to say it [the biggest impact on implementation] just had to be a collaboration of staff and teams working together because one person wasn’t able to do it all, you know, so we had our OT and recreation teams supporting to help with the visits and transport residents back and forth, so your own job was no longer just your job, it was everybody’s job just to help and make it happen … it definitely had to be a team effort because there was too much staff needed to make it all work. (Direct care staff, Resident Care Worker, Prince Edward Island)

## Discussion and Implications

We interviewed 54 LTC staff (32 implementation staff and 22 direct care staff) about their experience during the implementation of visitor support programs during the COVID-19 pandemic. Both implementation and direct care staff believed that family caregivers were essential to resident physical and mental well-being. The development and implementation of essential visitor programs will be especially informative during seasonal influenza outbreaks. Despite the essential nature of visitors, staff reported numerous challenges related to the program’s implementation. Using the CFIR domains and constructs as an analytic guide helped us to identify challenges and facilitators affecting implementation. We identified two broad themes (external innovation; organizational context) that describe both barriers and facilitators to implementing the visitor support program. Within these themes, CFIR constructs, including the intervention source (i.e., external) were related to barriers to implementation. Staff felt the visitor support program was an essential program; however, the externally mandated program had constantly changing policies and short timelines to develop processes and address changes. Although staff appreciated the ability to adapt the implementation process for their LTC home, the expectation for rapid change coupled with the top–down, hierarchical nature of the policy directives meant LTC homes did not have sufficient time to trial implementation processes (e.g., testing new visitor policies on one unit before moving to the rest of the facility). To expedite changes in response to changing regional epidemiology, health authorities notified the public at the same time as LTC homes, inhibiting LTC homes from getting input from residents, family members, and staff on how to facilitate the most positive outcomes. These findings have practical implications for communication processes within the health system. We need a defined process to allow bidirectional information flow between provincial ministries of health and health authorities and LTC homes. Direct care staff were not consulted during the development or implementation of the visitation program. Staff often did not have sufficient access to the knowledge and information, which is necessary to implement a given innovation. Lack of information meant that front line workers experienced practical and logistical issues in implementing the guidelines. Including end-users in the development and implementation planning process enhances care processes and service delivery. If LTC staff were consulted during design and implementation planning, some of these issues could have been proactively identified and the program elements modified to address the challenges reported by staff.

As identified in the CFIR, implementation was challenged by structural characteristics (i.e., LTC home space) and antecedent conditions such as available staff resources; some facilities lacked the space to accommodate safe visitations, and some did not have sufficient human resources to reallocate into essential new roles. Staff shortages were mitigated when one of the provinces (Nova Scotia) provided financial resources to hire additional direct care staff (Government of Nova Scotia, 2021). Our findings are consistent with recent research highlighting the reactive nature of COVID-19-related responses (Baral, 2021). Under-resourced health care settings do not have the capacity or resources to quickly pivot when external situations necessitate immediate action. LTC facilities are a particularly vulnerable sector because they are chronically under-resourced, with pervasive issues related to staff shortages, aging infrastructure, and variable education and training, particularly related to management and leadership (Estabrooks et al., 2020). Our findings add to the immense body of evidence calling for increased investment in the LTC sector for staffing and education, allowing LTC homes to be proactive and build sufficient capacity to carry out essential programs.

Our analysis identified several CFIR constructs, including communication, leadership, and teamwork, strongly influencing the implementation of visitor support programs within LTC homes. Many barriers (i.e., human and material resources, organizational readiness) and facilitators (i.e., supportive leadership and positive organizational culture) identified in our study, determine how quickly health care settings can implement new evidence, and whether implementation or innovation outcomes are positive (Piat et al., 2021; Thijssen et al., 2021; Xue et al., 2021). LTC homes with cultures supporting teamwork, strong leadership, and two-way communication with staff were more able to implement the frequently changing program. Effective staff communication facilitated implementation, but broken communication channels resulted in confusion between staff and residents, leading to an erosion of relationships and diminished likelihood of program success. This is particularly challenging because LTC homes often have a mixture of staff roles (e.g., regulated, unregulated, and allied), employment designations (e.g., full time, part time, and casual), and shifts (e.g., days, evenings, and nights). Communication among staff, particularly between administrators/managers (implementation staff) and direct care staff, is essential and needs to be resourced. Systems that support communication (e.g., unit huddles) can facilitate communication and foster engagement and a positive work culture. LTC homes with established clear communication processes and adaptive leadership styles prior to implementing the visitation program had a smoother transition (Corazzini et al., 2015; Xue et al., 2021). Existing communication channels backed by strong leadership led to early staff buy-in and increased staff motivation.

## Limitations

Our study was not without limitations. It was conducted during the COVID-19 pandemic, when attendant contextual stresses for LTC staff may have systematically or differentially affected people’s willingness to volunteer as participants, as well as their responses. Staff with particularly positive or negative feelings about the visitation program could have had affected responses. We conducted interviews in English; therefore, staff with English as an additional language may not be represented.

We included questions related to sex, gender, and other characteristics in our data collection and analysis; however, these factors did not emerge in our findings. We did find that LTC staff observed that residents had different experiences of support visitation based on their cognitive status. Our sample is relatively homogenous with respect to gender and race and ethnicity; which reflects the LTC workforce (predominantly female) and regional race and ethnic composition (predominantly White) but limits our ability to speak to issues related to inequities experienced by persons from marginalized groups. Other research in our team includes interviews with family members, rather than LTC staff, and may be able to better speak to issues related to inequities and the experience of family and residents.

Participation was defined at a facility level, which means that LTC homes with more interest in family visitation (and thus potentially better communication or implementation plans compared with nonvolunteer facilities) might be over-represented. Finally, we focused on facilitators and barriers to implementation processes and did not have an implementation outcome to distinguish the success or failure of the visitation program between facilities. Although we identified some actual implementation outcomes in our content analysis, our aim was not to examine causal factors on implementation or innovation outcomes. Causation coding may have allowed us to better discern causal chains between the CFIR constructs and implementation outcomes (Saldaña, 2021).

## Conclusions and Recommendations

Our study offers important insights into the experience of staff implementing visitor support programs in LTC homes during the COVID-19 pandemic. Communication between provincial governments and health authorities and LTC was limited, and facilities often learned about program changes along with the public. LTC staff were consistently in a reactive position rather than having adequate resources and time to proactively plan. More research is needed on the best practices for communication and collaboration between governmental organizations and LTC providers, particularly related to establishing proactive versus reactive planning strategies for future outbreaks of infectious disease.

We offer practical strategies for LTC homes and for provincial/state LTC decision-makers (see Supplementary Table 3). Strategies for LTC homes include supporting staff by providing more staff resources and increasing their recognition, and increasing the myriad communication approaches within facilities through technology and in-person communication. Strategies for provincial/state decision-makers should address the staff-implementation interface, because many directives were external to the homes, and their rational was not transparent. In addition, supporting infection prevention and control to allow the family to visit safely is needed, and recognizing the essential role families play in care is imperative.

Not all LTC homes had the same experiences. Contextual elements, including communication strategies, leadership, and staffing resources allowed some facilities to more effectively implement visitor programs and keep up with the constant change. Our study examined implementation staff and direct care staff who highlighted the importance of visitor programs for residents. Research from the perspective of residents on the impact of visitor programs on their quality of life is an essential area for future research.

## Supplementary Material

gnac175_suppl_Supplementary_Material

## Data Availability

There was no preregistration for this study. Data are available on request from the author.
