# Supplementary material for: With COVID Comes Complexity: Assessing the Implementation of Family Visitation Programs in Long-Term Care
Source: Gerontologist. 2022 Dec 3;63(3):490–500. doi: 10.1093/geront/gnac175 (PMC10028227; doi:10.1093/geront/gnac175)
Supplement: gnac175_suppl_Supplementary_Material [file gnac175_suppl_supplementary_material.docx]

**Supplementary Table 1.** *Consolidated Framework for Implementation Research CFIR Implementation elements*

| **CFIR Implementation Determinant Domains and Constructs** | |
| --- | --- |
| Domain | Constructs |
| 1. Intervention Characteristics | Intervention source  Evidence strength and quality  Relative advantage  Adaptability  Trialability  Complexity  Design quality and packaging  Cost |
| 1. Outer Setting Constructs | Patient needs and resources  Cosmopolitanism  Peer pressure  External policy and incentives |
| 1. Inner Setting Constructs | Structural characteristics  Networks and communication  Culture  Tension for change  Compatibility  Relative priority  Organizational incentives & rewards  Goals and feedback  Learning climate  Leadership engagement  Available resources  Access to knowledge & information |
| 1. Characteristics of Individuals Constructs | Knowledge and beliefs about the intervention  Self-efficacy  Individual stage of change  Individual identification with organization  Other personal attributes |
| 1. Process Constructs | Planning  Engaging  Executing  Reflecting and evaluation |
| **Antecedent Assessments^a^** | |
| Implementation climate parent construct moved to Antecedent Assessments from CFIR 1.0 determinants  Readiness for implementation parent construct moved to Antecedent Assessments from CFIR 1.0 determinants | |
| **Implementation Outcomes^a^** | |
| Anticipated (have not yet occurred) | Adoptability  Implementability  Sustainability |
| Actual (have already occurred) | Adoption  Implementation  Sustainment |
| **Innovation Outcomes** | |
| Impact on | Key decision makers  Innovation recipients  Innovation deliverers |

^a^ Changes to CFIR 1.0 based on Damschroder et al. (2022).

**Supplementary Table 2.** *Details and characteristics of participating LTC Homes*

| **Characteristic** | **Details from Participating LTC Home** |
| --- | --- |
| Province | 4 in Nova Scotia, 2 in Prince Edward Island |
| Owner-Operator Model | 2 Government, 2 Not-for-Profit, 2 For-Profit |
| Layout Design | 3 Neighbourhood, 3 Traditional |
| Year Built | 1971-2013 |
| Renovations over Time | Traditional LTC homes had minor revisions that ranged from the most extensive (increasing the number of private rooms or changes in line with infection prevention) to more minor mechanical or maintenance upgrades |
| Number of Residents | 36-120 |
| Number of Private Rooms | 36-120 (Neighborhood designs had the same number of residents to private rooms and Traditional had fewer private rooms than residents) |
| Family Council in LTC Home | 4 had a family council, 2 did not have a family council |
| Designated Caregiver | Descriptor of a designated family/friend in the province of Nova Scotia (4 LTC homes) |
| Partner in Care | Descriptor of a designated family/friend in the province of Prince Edward Island (2 LTC homes) |

*Note*. LTC = long-term care.

**Supplementary Table 3.** *Practice Considerations learned from implementation of visitation programs from staff perspectives*

| **DIRECTED TOWARDS LTC FACILITIES** | |
| --- | --- |
|  | |
| ***Actions to support staff working in LTC*** |  |
| Improve communication through multiple modalities, timing, and transparency   - Have regular in-person communication (i.e., huddles and team meetings) paired with memos, emails, and social media. - Be transparent - staff need to know the reason for rule changes and whether they were provincially-, or facility-driven. | |
| Supplement Staffing Resources   - Increase staffing resources—a dedicated coordinator for the visitation program, more long-term care assistants, and volunteers to support staffing and resident’s needs. - Develop or continue formal relationships with other organizations such as hospice, end of life care specialists, churches, and volunteers to support spiritual and end of life care. LTC homes that had pre-existing relationships and support from these organizations were often halted due to lockdowns. | |
| Increase Recognition and Support for Staff   - More training and education when changes are made - Recognition or a reward program for staff whose work is undervalued. - Support mental health of staff and family through regular check ins, interventions to mitigate stress, opportunity for formal support. | |
| ***Communication within the facilities*** |  |
| Recognize how communication and technology can be a barrier or enabler to implementation.   - Understand technology can be used to facilitate communication but should not replace in-person guidance for staff and families. | |
| Including families and direct care staff in meaningful ways.   - Know that scheduled discussions on virtual platforms can support family involvement. - Incorporate formal processes for input from direct care staff and families. | |
| **DIRECTED TOWARDS STATE/PROVINCIAL HEALTH AUTHORITIES** | |
|  | |
| ***Address staffing implementation interface*** | |
| Many of the suggested changes from staff were outside of the individual LTC home’s control and are addressed to the broader provincial health directives: | |
| More transparent and timely communication with public health.   - Consider giving LTC homes information prior to public announcements allowing them time to implement any changes to the program - Provide further information on how to implement the directive and any changes. | |
| Provide further staffing resources:   - Funding to hire more staff. - Increase wages for existing staff. - Have a system in place to provide emergency staffing to LTC homes when it is needed | |
| ***Broader considerations to address family, staff, and LTC home needs in the implementation process*** |  |
|  |  |
| Recognize family play a vital role providing care and emotional support to residents.   - Review protocols with a person-centered and family-centered lens. |  |
| Consider how blanket approaches are not best practice and how the program might impact different groups.   - Visiting hours are accessible and acknowledge DCG proximity to LTC home and working schedule. - Rules support persons living with dementia and other complex health conditions. - Considerations for larger families who have added pressure of choosing DCG. |  |
| Utilize knowledge gained during the COVID-19 pandemic to inform the implementation of visitation programs during future infectious disease outbreaks.   - Ensure proper infection prevention and control allowing family/friends and DCGs to visit safely. |  |

*Note*. LTC = long-term care; DCG = Designated Caregiver.

**Supplementary Figure 1**. *Timeline of Nova Scotia and Prince Edward Island LTC Public Health Directives during COVID-19 Wave 3 (2021)*


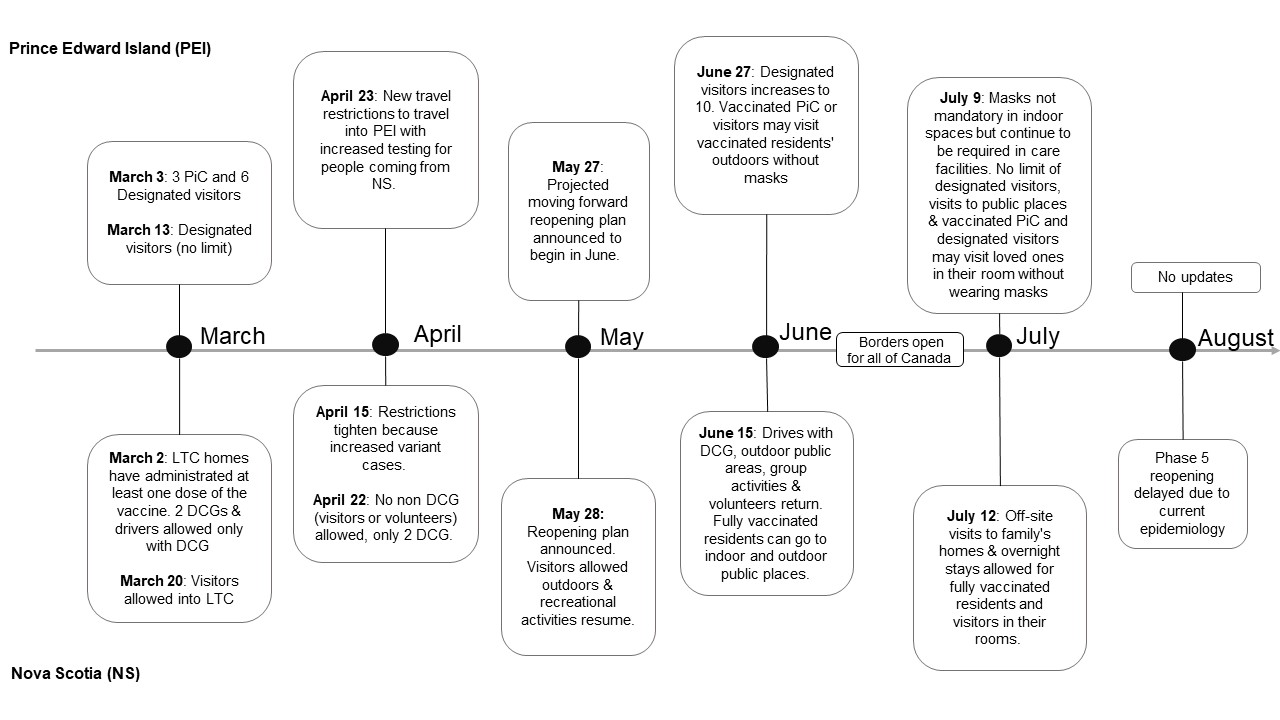


*Note*: PiC = Partner in Care; DCG = Designated Caregiver.

**Semi-Structured Interview Guide**

**Interview Guide with staff responsible for implementation**

**Participant ID: ____________________**

**Interviewer: ________________________________ Date: ___________________**

**Video □ Telephone □**

**Start time: ________________ Finish time: ______________**

OPENING AND CONSENT PROCESS:

[study introduction]

**Instructions for Interviewer re Consent:**

[obtain informed consent]

SECTION 1: BACKGROUND QUESTIONS – INDIVIDUAL AND ORGANIZATIONAL

*To begin, I would like to learn about your position at [INSERT FACILITY NAME].*

1. What is your position at [**INSERT FACILITY NAME**]?
   1. To whom do you report?- Please report their title/position as opposed to their name
2. How long have you been working here?
   1. How long working in this position?
3. What are your main responsibilities?
4. What have been your responsibilities/role in relation to the implementation of the Designated Family Caregiver program (NS)/Partner in Care program (PE) program?

*Now I am going to ask you general questions about the place where you work.*

Can you describe how [**INSERT FACILITY NAME**] acted upon the directive to introduce the Designated Family Caregiver program (NS)/Partner in Care program (PE). What was the sequence of events as you recall?

What type of supports were available? Was that sufficient?

How much input did you/others have?

To what extent was family input obtained/considered? Why or why not?

In what ways was the approach taken similar or different to introducing new initiatives related to family or friend caregivers? (e.g., type/amount of support, who is responsible, who provides input).

SECTION 2: GETTING INVOLVED WITH IMPLEMENTATION

*These next questions are about getting involved with the implementation of* Designated Family Caregiver program (NS)/Partner in Care program (PE). *When answering these questions I would like you to think from the perspective of your role in implementing the* Designated Family Caregiver program (NS)/Partner in Care program (PE) *program.*

1. How did your facility become involved in the implementation?
2. How did you personally get involved?
3. Would you say implementing the Designated Family Caregiver program (NS)/Partner in Care program (PE) was more externally driven (i.e. you felt there was a directive from someone else to participate), or was it internally motivated (the facility felt a need for the program)? Why?
   1. Did you or someone at your facility have a say in whether you implemented the Designated Family Caregiver program (NS)/Partner in Care program (PE)?
   2. Did you feel pressured to introduce the program? By whom? Why?
4. Prior to implementing the program did you see a need for this type of program? Why or why not?
5. Do you feel that by being part of the implementation you are contributing to best practices [Definition of best practices if requested by participant: Best practices are a set of guidelines or ways of doing things that represent the most appropriate course of action in a given situation. For example, providing a resident who is at risk of falling with hip protectors].?
6. Can you describe any planning you were involved with to get the initiative started?
7. Who was involved in the planning process?
8. Do you think the appropriate people were involved in the planning process? If not, who would be an appropriate person and/or team to get involved? To what extent were family involved?

**Prompt (if relevant):** what were the consequences of not having the appropriate people involved it the planning process?

1. Were efforts made to elicit feedback from residents and family about the implementation?
2. Do you see a particular resident need that would be (or is being) met by implementing this program?
   1. How do you think the program is a need? What kind of feedback do you get from residents? Families?
   2. What outcomes do you hope for? **Probe:** resident quality of life, social interaction, physical care needs.
3. What supports were available to get the program started? Was that enough or would you require less or more support in the future?

SECTION 3: IMPLEMENTATION EXPERIENCES

*Now I will ask you some questions about your experience trying to implement the program and the type and level of support you had.*

1. On a scale of 1-10, with 1 being very easy and 10 being nearly impossible, how difficult has it been to implement Designated Family Caregiver program (NS)/Partner in Care program (PE)? Why do you rate the program at this level?
2. Please describe any circumstances, if any, in which the program was very difficult to implement.
3. What barriers did you encounter in trying to implement the program?
   1. Within the facility
      1. **Probe:** number of staff, staff composition, staff motivation and beliefs, size of facility, physical space, access to PPE, development of training/protocols for families, communications
   2. Outside limitations such as resources, bureaucracy or training
4. What were the facilitators you experienced in trying to implement the program?
   1. Within the facility
      1. **Probe:** staff resources, physical space, size of facility
   2. Outside facilitators such as resources, bureaucracy or training
5. What had the biggest impact on your ability to implement the program?
6. How do you think the culture of your organization influenced the implementation? **Probe:** Does the staff often work as a team, had success implementing other initiatives, leaders open to ideas,
7. What type of feedback did you receive on how implementation was going?

If you did receive feedback…

1. Who do you receive feedback from?
2. How was it used?
3. Was it discussed with others?

If you did not receive feedback

1. What type of feedback would be useful to implement?
2. How might this lack of feedback negatively impacted implementation?
3. Who was especially instrumental in helping to get the Designated Family Caregiver program (NS)/Partner in Care program (PE) implemented? (you can note more than one person if needed) (can be inside/outside facility)
4. What roles did these people play? What was their position in the program?
5. Were they formally appointed in this position, or was it more of an informal role?
6. Did they help persuade people who may have initially been indifferent or resistant to the program?
7. What level of involvement do leaders in your organization have with the program?
8. What aspects of the program would you have liked to adapt to fit the routines within your facility?
9. Is there anything you or your organization have learned from implementing this initiative that might be helpful for future implementation initiatives at [**INSERT FACILITY NAME]?** What about lessons learned for others to implement in NS/PE or elsewhere?

SECTION 4: COSTS/AVAILABLE RESOURCES

*Now I will ask you some questions about the cost of the program and the available resources you had when implementing the program.*

1. Did your facility have adequate funding to support implementation of this program?]
   1. If no, in what area(s) would more funding have been required?
2. Can you think of any incentives that could influence implementation of a Designated Family Caregiver program (NS)/Partner in Care program (PE)?
   1. If yes, how?
3. Do you think there are adequate supports/resources to keep the Designated Family Caregiver program (NS)/Partner in Care program (PE) going during the duration of pandemic (i.e., likely fall of 2021)?
   1. What types of follow-up support would be needed? (e.g. facility facilitation, monetary, facility help, administrative time …)
   2. Does your facility have consistent resources to continue to support implementing a Designated Family Caregiver program (NS)/Partner in Care program (PE)? What resources do you have that will be helpful?
   3. How would additional supports or resources make a difference? What would these supports look like?
4. What do you think about the implementation guide that was created?
   1. Do you think it is helpful for facility? Any suggested changes in content or format?

SECTION 5: IMPACT/OUTCOME

*Now I would like you to think about the impact or effect this program has had at [INSERT FACILITY NAME].*

1. To what extent do you think the Designated Family Caregiver program (NS)/Partner in Care program (PE) is meeting its goals
2. What have these support visitations meant for [INSERT FACILITY NAME]? What has been the impact/benefit to:

**Probe:** Positive & negative outcomes/impacts (e.g., reduced resident loneliness, access to additional personal care/support from family [feeding, mobilizing]) . If there were changes over time how have they been both positive & negative.

- 1. Residents
  2. Family
  3. Staff

1. Are there some people/residents who seem to benefit more from the program than others? Why might this be?

**Probe**: Differences based on age, gender, sexuality, economics, ethnicity/culture, language, family dynamics

1. Have you noticed that implementation has had differential (or varying) impacts on different families and residents depending on their cultural/economic background, family structure or proximity to family? For example, do you notice that some family members who were previously frequent visitors have not made full use of the DFC/PIC program?
   1. If so, why do you think this is?

SECTION 6: WRAP-UP

*These next few questions are geared towards networking with other organizations around the program and collecting some general information about you.*

1. Are there other organizations that have been or would be helpful to network with to provide you with insights on how you could improve the implementation?
   1. If so, which ones? What have you/would you benefit from?

*And before wrapping up the interview, I would like to get some additional information about you...*

| 1. Can you please tell me what year you were born? |  |
| --- | --- |
| 1. What is your gender? (e.g. man, woman, trans, non-binary or another term) |  |
| 1. What is the highest level of education you have completed? |  |
| 1. In what country were you born? |  |
| 1. To what ethnic group do you identify? |  |
| 1. In addition to English, what other languages do you speak? |  |

*We are on the last set of questions.*

1. Do you have any final suggestions for improving the program and its implementation?
2. Is there anything else you'd like to add in terms the fairness of the policy implementation or how it might impact different groups of people differently?

**Probe**: Differences based on age, gender, sexuality, economics, ethnicity/culture, language, family dynamics

That concludes my questions. Is there anything I’ve missed or anything else you’d like to add?

[Stop recording]
